# Detection of phosphatidylethanol after ethanol intake with targeted blood alcohol concentrations of 0.6 g/kg and 0.75 g/kg

**DOI:** 10.1007/s00414-024-03379-w

**Published:** 2024-12-04

**Authors:** Franziska Spleis, Matthias Bantle, Dominik Schuldis, Lorenz M. Bell, Annette Thierauf-Emberger, Wolfgang Weinmann

**Affiliations:** 1 Institute of Forensic Medicine, Medical Center - University of Freiburg, Albertstraße 9, 79104 Freiburg, Germany; 2 Faculty of Medicine, University of Freiburg, Freiburg, Germany; 3 Institute of Forensic Medicine, Forensic Toxicology and Chemistry, University of Bern, Bern, Switzerland

**Keywords:** Ethanol, Phosphatidylethanol (PEth), Alcohol consumption, Alcohol markers, PEth sensitivity, Abstinence

## Abstract

Alcohol consumption is widespread in most western countries such as Germany and a relevant risk factor for morbidity and mortality. Sensitive detection of alcohol consumption using suitable markers is therefore of central importance for clinical and forensic diagnostics. Direct alcohol markers are non-oxidative products of ethanol, which are produced in the body during the degradation of ethanol and provide high sensitivity and specificity. Phosphatidylethanol (PEth) is a promising marker for detecting alcohol consumption in the past days to weeks. The aim of this study was to determine the minimum amount of ethanol for a single alcohol consumption that leads to a detectable increase in blood PEth concentration. Therefore, 12 participants were recruited and, after four weeks of abstinence, drinking tests were carried out with target blood alcohol concentrations (BAC) of 0.6 g/kg and 0.75 g/kg. The PEth samples were obtained as dried-blood spots on the test day and the three following days and analyzed using LC-MS/MS. The result of the study were a detectable increase of PEth in the blood above limit of detection after both drinking events in all participants and an increase in PEth above the cutoff concentration for abstinence of 20 ng/mL in 9/12 (75%) and 7/12 (58%) participants, respectively, from a minimum BAC of 0.48 g/kg. These results make PEth appear promising as a marker for controlled moderate alcohol consumption.

## Introduction

Excessive alcohol consumption is a widespread problem worldwide, but especially in Europe [1, 2]. Besides the numerous health consequences, drunk driving is a major problem in terms of traffic safety. Because of the numerous health consequences, screening procedures are recommended for the detection of high-risk alcohol consumption [3]. Proof of alcohol abstinence also plays an important role, for example in context of organ transplantation or traffic-related issues. In Germany, for example, it is necessary to prove 6 or 12 months of abstinence in order to regain a driver’s license after losing it due to alcohol intoxication. Abstinence monitoring can be provided by means of suitable alcohol consumption markers.

Alcohol consumption markers are substances in the body that are used to detect and assess acute (hours to days) or chronic (weeks to months) alcohol consumption and can be divided into indirect and direct markers. While indirect alcohol markers such as mean corpuscular volume of erythrocytes (MCV), gamma glutamyl transferase (GGT) or carbohydrate-deficient transferrin (CDT) indicate the influence of alcohol on specific organ systems in the body, direct alcohol markers such as ethyl glucuronide (EtG) and ethyl sulfate (EtS) are non-oxidative products of ethanol, which are produced in the body during the degradation of ethanol. Due to their higher sensitivity and specificity, direct alcohol markers are more suitable than indirect alcohol markers for abstinence monitoring [4–7].

Phosphatidylethanol (PEth) is a promising direct alcohol marker that has become increasingly relevant in recent years [6, 8–11]. PEth is an abnormal phospholipid and is formed from phosphatidylcholine on cell membranes by the enzyme phospholipase D (PLD) in the presence of ethanol [12, 13]. Up to present day, 48 different PEth homologues are known, defined by the combination of different fatty acids bound to the glycerin backbone. The two most commonly found PEth species in the body, PEth 16:0/18:1 and PEth 16:0/18:2 [14], account for approximately 60% of total PEth [15]. With a half-life of around four days [16, 17], PEth can generally be detected in blood for several days to weeks [17] but exhibits a large interindividual variance [18]. According to Schröck et al., PEth 16:0/18:1 can be detected for three to twelve days after a single consumption of alcohol that led to a BAC of 1.0 g/kg [19]. In other studies, PEth even was detected after a single consumption of alcohol concentrations that led to BAC between 0.25 and 0.8 g/kg [20, 21]. PEth can be used in a variety of ways in clinical and forensic settings, both to identify risky drinking habits and to detect minor or one-off drinking events. Recently, the use as an abstinence marker for assessment of driving aptitude has also become possible. Since 2022, PEth with a threshold of 20 ng/mL can be used alongside the detection of EtG in urine and hair as a proof of abstinence for regaining a driver’s license in Germany [22].

Thresholds for interpretation of PEth values in the forensic or clinical field have been suggested by Ulwelling and Smith [23] and later by the society of PEth research [24]. A cut-off of 20 ng/mL PEth 16:0/18:1 has been suggested for differentiation between abstinence (or very low alcohol consumption) and moderate drinking in both publications.

The aim of this study was to narrow down a minimum amount of ethanol consumption that leads to the formation and mass-spectrometric detection of PEth in blood after abstinence and PEth concentrations above the cutoff of 20 ng/mL. For this purpose, after four weeks of abstinence, two consecutive drinking tests were carried out with target blood alcohol concentrations of 0.6 and 0.75 g/kg. Previously published studies indicate a possible consumption range, but mostly show an insufficient abstinence phase which led to increased baseline values at the start of drinking event [19–21]. The findings of this study contribute to the discussion of PEth as a possible marker for the detection of single drinking events.

## Materials and methods

### Participants

Twelve volunteers were recruited at the University of Freiburg, Germany to take part in the study. Inclusion criteria was an age of at least 20 years and a good physical constitution. Exclusion criteria for participation were the presence of pregnancy or serious illnesses such as cardiovascular, respiratory, metabolic, liver or kidney disease, mental or neurological illnesses or addiction. The presence of diseases was recorded by questionnaire at the beginning of the study. Serious illness led directly to exclusion from the study. To screen for alcohol abuse, the average alcohol consumption per week was surveyed and the indirect alcohol markers GGT and MCV were determined. Also a single analyzes of the direct alcohol marker PEth was also conducted. Participation was voluntary and the participation in the study could be cancelled at any time. Table 1 contains data of the participants.

**Table 1:** Descriptive data of the participants

No.	Sex	age [years]	Height [cm]	Weight [kg]	BMI [kg/m^2^]
1	m	27	183	75	22.4
2	m	25	180	78	24.1
3	m	27	180	70	21.6
4	m	25	194	94	25.0
5	f	27	174	68	22.5
6	f	24	175	57	18.6
7	f	22	165	57	20.9
8	m	23	191	88	24.1
9	f	24	165	53	19.5
10	f	23	170	60	20.8
11	m	25	172	71	24.0
12	f	25	180	65	20.1
Mean		24.75	177.42	69.67	21.96
(SD)		(1.59)	(8.76)	(12.06)	(1.96)

### Experimental design

**Fig. 1:**
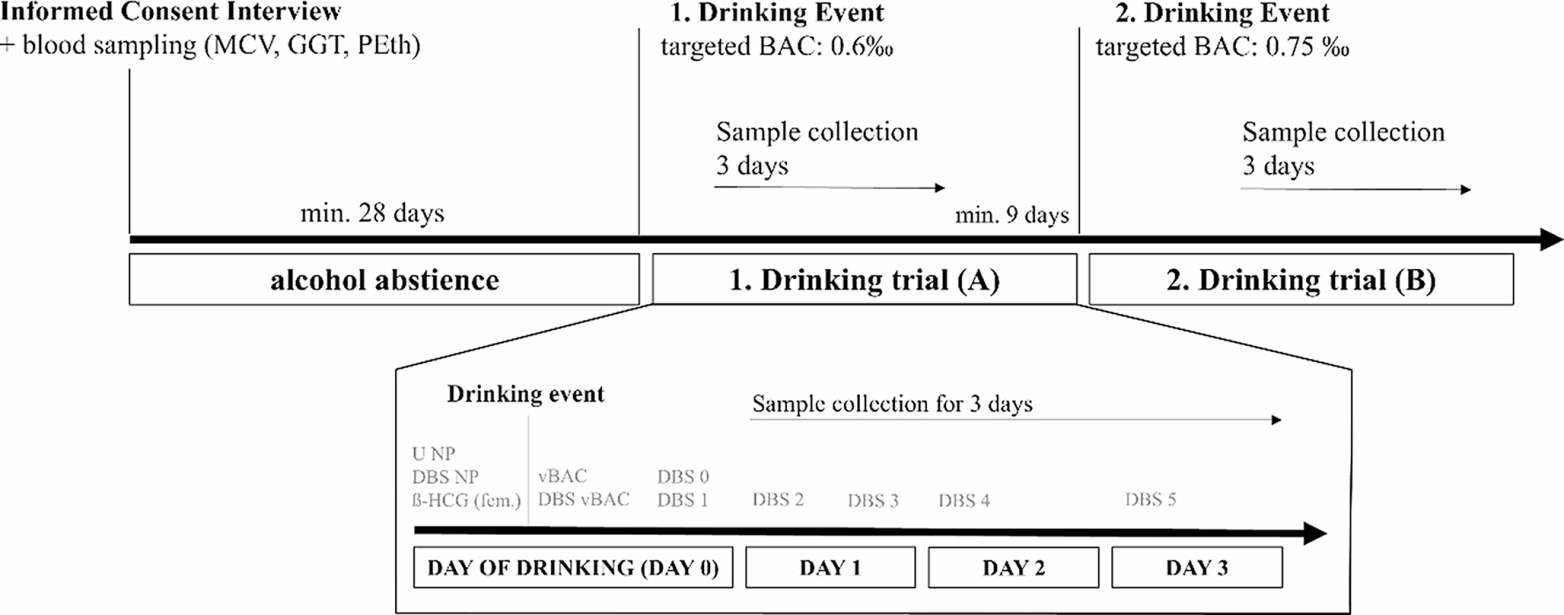
Time schedule and sample collection. U: Urine sample, NP: Sample before start of drinking, DBS: Dried blood spot, vBAC: venous blood alcohol concentration

The drinking study took place at the Institute of Forensic Medicine at the University Medical Centre Freiburg from April to July 2023. Before the start of the trial, all volunteers were interviewed by a doctor and informed about possible risks. An initial sample collection was done (as explained below). All participants underwent a four-week abstinence phase, followed by two drinking trials (trial A and B) with targeted blood alcohol concentrations (BAC) of 0.6 g/kg and 0.75 g/kg and subsequent three-day sample collection in a private setting. There was a minimum period of nine days between the two drinking trials. The time schedule of the trial is shown schematically in Fig. 1. The individually required amount of alcohol (Wodka Gorbatschow, 37.5vol%, mixed with fruit juice) was calculated using the Widmark formula and was consumed within 30 min (1st drinking event) or 45 min (2nd drinking event). The participants’ last meal was consumed no later than two hours before the start of the drinking event. Once the target BAC was reached, the participants were kept under medical supervision at the Institute of Forensic Medicine until a breath alcohol concentration of less than 0.05 mg/L was reached.

### Sampling

Venous blood samples were collected at the beginning of the abstinence phase, to identify (and potentially exclude) participants with alcohol abuse by analyzing MCV and GGT as well as a single determination of a baseline value for PEth as a dried-blood spot sample (DBS) and, if necessary, follow-up checks after 3 weeks. The determination of PEth from capillary blood of the fingertip and dried on a filter paper card as DBS was carried out due to the simplified storage and sample collection. The result accuracy of DBS is comparable to venous blood samples [5, 25, 26]. DBS sampling is easier to handle during the trial, as it can be done by self-sampling by the volunteers “at home” on the days after the supervised drinking day. DBS samples were collected on DBSV cards (Greencheck DBSV, Protzek, Lörrach, Germany). In addition, before the start of both drinking trials, urine samples were collected for determination of EtG by LC-MS/MS with a limit of detection (LOD) of 100 ng/mL to confirm abstinence prior to the experiment. Women took a pregnancy test (*β-HCG*) before consuming alcohol. In each of the two drinking trials, one urine sample was taken to determine EtG, seven capillary blood samples were taken as DBS to determine PEth and one whole blood sample was taken to determine the BAC 45 min after the end of drinking. On the day of the drinking event (later referred to as “day zero”), a urine sample (*U NP*) and a DBS sample (*DBS NP*) were taken before the start of the drinking test. Breath alcohol was monitored at 15-minute intervals from 45 min after the end of drinking on throughout the study day until it fell below an AAC of 0.05 g/l. A venous whole blood sample (*vBAC*) was obtained 45 min after the end of drinking to determine the BAC, from this sample a DBS (*DBS vBAC*) was prepared. Further DBS from capillary blood were taken 30 min after the breath alcohol concentration fell below 0.05 g/l (*DBS 0*) and in the evening at around 9 pm (DBS 1) by self-sampling in a private setting without supervision. In addition, four capillary blood DBS samples were taken by the participants on the three following days, one in the morning and one in the evening on day 1 (*DBS 2–3*) and one in the morning on day 2 (*DBS 4*) and on day 3 (*DBS 5*). Figure 1 provides an overview of the sample collection. The schedule described in detail for the first trial was also applied for the second trial.

### Analytical

All solvents used were of HPLC quality. Methanol (MeOH) (≥ 99.9%) was obtained from Biosolve BV (Valkenswaard, Netherlands), acetonitrile (MeCN) (99.9%) from Acros Organics (New Jersey, USA) and isopropanol (≥ 99.5%) from Fisher Scientific (Loughborough, UK). Ammonium acetate (Fractopur) was purchased from Merck (Darmstadt, Germany). Sigma-Aldrich (Buchs, Switzerland) provided formic acid (FA) (50%).

PEth 16:0/18:1 was analyzed on DBS by LC-MS/MS as described elsewhere [26, 27]. This type of DBS card system was earlier compared to classical DBS cards [28]. In brief, for each sample, one paper tooth was detached from its holder and placed in a 2 mL plastic vial (Sarsted, Nümbrecht, Germany). MeOH (1 mL) and pentadeuterated internal standard solution (10 µL) were added. The samples were then shaken for 20 min (IKA Vibrax, Staufen, Germany) and subsequently centrifuged for 10 min at 13,000 rpm and 8 °C (Mikro 220, Hettich, Tuttlingen, Germany). Then, the extract was transferred to 2 mL auto sampler glass vials (Wicom, Heppenheim, Germany) and evaporated at 50 °C under a gentle stream of N_2_. After reconstitution with MeOH (100 µL), the sample (injection volume 4.5 µL) was analyzed using an LC-MS/MS system consisting of an UltiMate^®^ 3000 HPLC system (Dionex, Thermo Scientific Instruments, Reinach, Switzerland) coupled to a 5500 QTRAP with a TurboIonSpray source (Sciex, Toronto, Canada) operated in negative ionization mode using a previously published method [26]. The limit of quantitation (LOQ) was 10 ng/mL, LOD was set at 5 ng/mL, and calibration ranged from 7.5 to 1500 ng/mL.

EtG as well as EtS in urine were analyzed by LC-MS/MS as described by Luginbühl et al. [29]. The limit of quantitation was at 0.1 mg/L. In short, 150 µL of working solution (300 µL EtG-D_5_ (0.1 mg/mL, Cerilliant, Round Rock, Texas, USA) and 50 µL EtS-D5 (0.1 mg/mL, Lipomed, Arlesheim, Switzerland) in 25 mL MeCN) was added to 50 µL of urine sample, which was then subsequently shaken for 5 min and centrifuged for 10 min at 8 °C and 13,000 rpm. 100 µL of supernatant were transferred to an auto sampler glass vial and evaporated at 50 °C under a stream of N_2_. After reconstitution in 100 µl water/ MeCN (95/5) with 0.1% FA, the sample was analyzed using an LC-MS/MS system consisting of an UltiMate ^®^ 3000 HPLC system (Dionex, Thermo Scientific Instruments, Reinach, Switzerland) coupled to a 3200 QTRAP instrument (Sciex, Toronto, Canada) operated in negative ionization mode using a previously published method [29]. LOQ was 0.1 mg/L, and calibration ranged from 0.1 to 10 mg/L.

The determination of the blood alcohol concentration (BAC) from serum was carried out at the Institute of Forensic Medicine at the Freiburg University Medical Centre using headspace gas chromatography with flame ionization detection (HS-GC-FID). After centrifugation of the blood samples, 100 µL supernatant with 500 µL tert-butanol solution (internal standard) was pipetted into a 20 mL headspace vial. The analysis was performed on two GC-FID systems: A Clarus 580 with an Rtx^®^-BAC Plus 1 column and a Clarus 680 with an Rtx^®^-502.2 column, both coupled to TurboMatrix headspace samplers (Perkin Elmer, Rodgau, Germany).

## Results

The results of the venous blood tests at the beginning of the study were all within normal physiological ranges, all pregnancy tests of the female participants and the urine samples for EtG and EtS (*U NP*) before the start of the drinking events were negative.

Measured BAC 45 min after finishing the first drinking event varied between 0.47 g/kg and 0.68 g/kg with a mean value of 0.56 g/kg, the second drinking reached BAC between 0.53 g/kg and 0.87 g/kg (mean 0.67 g/kg). The achieved BAC and PEth 16:0/18:1 concentrations are shown in Table 2.

**Table 2:** BAC and PEth 16:0/18:1 concentrations

No.^a^	Calculated amount of alcohol [g]	BAC [g/kg]	Baseline PEth^b^ [ng/mL]	max. PEth [ng/mL]	PEth DBS 2 (day 1) [ng/mL]	PEth DBS 5 (day 3) [ng/mL]
1 A	55.5	0.64	16.1	28.6	21.5	16.5
1B	67.5	0.75	16.5	44.9	32.9	26.6
2 A	59.3	0.57	< 10	23.7	13.6	< 10
2B	72.4	0.73	< 10	33	23.0	17.2
3 A	51.8	0.48	< 10	20.7	15.2	< 10
3B	63.0	0.69	< 10	19.8	17.7	16.3
4 A	69.4	0.68	< 10	24.6	17.4	16.5
4B	84.8	0.87	16.5	28.4	25.6	23.8
5 A	43.1	0.61	15.3	21.2	13.9	11.8
5B	52.5	0.66	11.8	17.5	17.4	19.8
6 A	39.0	0.47	< 10	12.3	< 10	< 10
6B	47.6	0.65	< 10	15.9	15.9	11.2
7 A	36.0	0.52	< 10	22.1	< 10	< 10^c^
7B	44.3	0.53	< 10^d^	22.5	15.0	11.4
8 A	64.9	0.55	< 10	15.5	15.5	< 10
8B	79.5	0.55	< 10	23.1	23.1	16.8
9 A	33.4	0.50	< 10	21.1	11.7	11.8
9B	40.9	0.61	11.8	19.9	15.3	< 10
10 A	37.9	0.58	< 10	24.6	16.1	11.0
10B	46.5	0.53	11.0	19	12.9	15.4
11 A	52.5	0.55	< 10	18.1	17.9	13.2
11B	64.1	0.78	13.2	28.4	23.1	22
12 A	43.9	0.51	< 10	28.8	22.6	19^c^
12B	54.4	0.63	19^d^	38.1	19.4	10.2^c^

^a^ Number of volunteer, A: first trial, B: second trial

^b^ for the 1st drinking event: Initial PEth concentration during the abstinence phase. For the 2nd drinking event: PEth concentration of the last day of 1st drinking event, except for participants 7 and 12: PEth concentration from day 2 of 1st drinking event (see “d”)

^c^ PEth concentration taken from day 2 (DBS 4) due to sampling problems on day 3

In both drinking tests, the formation of PEth above the LOD of 5 ng/mL was detected in all subjects. The maximum values for PEth were above the cutoff concentration for abstinence of 20 ng/mL in 9/12 (75%) participants in the first drinking trial and ranged up to 28.8 ng/mL with a mean of 22.18 ± 3.98 ng/mL. In the second drinking trial, 7/12 (58%) participants were above the cutoff up to a maximum of 44.9 ng/mL with a mean of 25.98 ± 8.50 ng/mL. All BAC above 0.7 g/kg led to PEth concentrations > 20 ng/mL, the lowest BAC that exceeded the cutoff concentration was 0.48 g/kg. The lowest amount of alcohol that led to a PEth > 20 ng/mL was 33.0 g. The maximum PEth concentrations were reached in 13/24 (54%) of cases in the DBS vBAC. For the remaining participants, the maximum was reached during day 0 (6/24) or on day 1 (5/24). PEth concentrations declined rapidly in the subsequent samples. In the first trial, all values were < 20 ng/mL on day 1, except for one participant. In the second trial, the concentration declined slower: on day 3, three samples were > 20 ng/mL and all samples except one were > LOQ of 10 ng/mL.

A few individual PEth values were removed from the analysis due to implausible results and are discussed below. The relevant values for this publication are listed in Table 2.

## Discussion

The aim of the study was to determine the minimum amount of ethanol that leads to an increase in the alcohol marker phosphatidylethanol after a single alcohol consumption. After two drinking events with targeted BACs of 0.6 g/kg and 0.75 g/kg and BAC measured at specific points with mean values of 0.56 g/kg and 0.67 g/kg, PEth 16:0/18:1 was analyzed over a period of four days and the degradation was tracked. The result of the study was a detectable increase in PEth in the blood > LOQ of 10 ng/mL after both drinking events in all participants and an increase in PEth above the cutoff concentration for abstinence of 20 ng/mL in 9/12 (75%) and 7/12 (58%) participants, respectively, from a minimum BAC of 0.48 g/kg.

The formation of PEth in a majority of participants with concentrations above 20 ng/mL after consumption of ethanol with an aimed concentration of 0.6 g/kg or 0.75 g/kg is consistent with previous studies. In a recent study, Herzog et al. found that, starting from baseline PEth concentrations in a similar range as in this study, alcohol consumption leading to a BAC of at least 0.6 g/kg yielded average PEth 16:0/18:1 concentrations of 29.1 ng/mL with 2/3 of the participants achieving maximum concentrations above 20 ng/mL. A fast decline was also observed in this study. Instead of capillary dried blood spots, in the study by Herzog et al., venous blood samples were collected and applied onto DBS cards [30]. In similar studies, the formation of PEth was already detected in measured BAC from 0.25 g/kg [20]. Hill-Kapturczak et al. were also able to demonstrate an increase in PEth after consumption of 0,4 g/kg and 0.8 g/kg ethanol [21]. One reason for the PEth formation at lower alcohol levels in these previous studies may have been the elevated baseline due to a short abstinence period and a low LOQ of 5 ng/mL chosen. On the other hand, Stöth et al. found no formation of PEth above 20 ng/mL at measured BAC up to 0.63 g/kg [31]. The strength of our study lies above all in the four-week abstinence phase, during which baseline concentrations for PEth between 10 and 20 ng/mL (9/24) and below the LOQ (15/24) could be determined in all participants.

As shown above, the cutoff concentration of 20 ng/mL was exceeded by more participants in the first drinking event than in the second drinking event. These results are contrary to the expectation of a dose-dependent increase in PEth after alcohol consumption, which has been demonstrated in numerous studies [32, 33]. A possible explanation for this may be the expanded measurement uncertainty (k = 2) of the laboratory method of 29.2% and also individual factors of the participants on the individual test days [34, 35]. Large interindividual differences in PEth formation have already been described in numerous studies [18–21, 36]. Another influencing factor is the variance in the achieved BAC, which was significantly lower than the target BAC for some participants. The mean BAC was 0.56 g/kg in the first drinking trial and 0.67 g/kg in the second, both below the targeted BAC. For instance, participant 10 exhibited a lower BAC and a lower maximum PEth concentration during the second drinking event compared to the first, despite consuming more alcohol. However, higher maximum PEth concentrations were achieved on average in the second drinking trial, correlating with the higher BAC. The detectability of PEth above the cutoff concentration was also longer for the second drinking trial: 3 days versus only 1 day after the first drinking trial.

In comparison to previous trials by our working group, requirements regarding food intake were made to prevent a delay of alcohol absorption in the intestine [31]. As already pointed out, the long abstinence phase is another strength of this study with baseline values < LOQ. Furthermore, negative EtG concentrations in urine samples collected before each drinking event for all participants also support the reported abstinence during the days before the drinking trial. An important limitation of this study is the small and homogeneous study population, resulting in limited external validity. Further studies with a larger sample of participants are desirable to confirm the findings. Nevertheless, an important insight into the estimation of the minimum consumption quantity for the formation of PEth was gained.

Baseline concentrations (at the beginning of the abstinence phase) were all below 20 ng/mL or even below 10 ng/mL (LOQ). Due to the rather short time of approximately 10 days of abstinence between the first and the second trial, the last sample of the first trial was taken as “baseline concentration” for the second trial. However, for volunteer 7 and 12, this was already the DBS sample of day 2 of the first trial, instead of day 3, due to sampling problems with the last samples.

DBS samples DBS 2 to DBS 5 were collected “at home” by participants as “self-sampling”. Even though the participants were instructed on how to correctly collect a capillary blood sample, this procedure could present a potential risk with respect to PEth concentrations: If too much pressure is applied onto the finger, the blood might be diluted leading to a falsified result [37]. The analysis of the hematocrit value might help to address this issue [38].

In this study, we did not include a placebo group, which we found not necessary due to the fact that we have used DBS samples from reliable teetotalers or long-term abstainers for many years, and never found a positive PEth concentration in those samples.

## Conclusion

In this study, the formation of phosphatidylethanol above the quantification limit of 10 ng/mL was detected in all participants with a BAC between 0.47 and 0.68 g/kg. The minimum BAC that resulted in a PEth concentration above a cut-off of 20 ng/mL was 0.48 g/kg. These results make PEth appear promising as a marker for controlled moderate alcohol consumption. Its use as an abstinence marker for regranting of the driver’s license based on detection after a single alcohol consumption also appears possible. However, the short detection period of one to three days and the moderate amount of alcohol that led to an increase in PEth in this study mean that further research into the detection period in a larger group of subjects is needed for a definitive assessment.

## Data Availability

Data underlying this article will be shared on reasonable request to the corresponding author.

## References

[1] Griswold MG, Fullman N, Hawley C, Arian N, Zimsen SRM, Tymeson HD et al (2018) Alcohol use and burden for 195 countries and territories, 1990–2016: a systematic analysis for the global burden of Disease Study 2016. Lancet Lond Engl 392:1015–1035. 10.1016/S0140-6736(18)31310-2 10.1016/S0140-6736(18)31310-2 PMC6148333 30146330

[2] World Health Organization (2019) Global Status Report on Alcohol and Health 2018. World Health Organization, Geneva

[3] Deutsche Gesellschaft für Psychiatrie und Psychotherapie, Psychosomatik und Nervenheilkunde (DGPPN) Deutsche Gesellschaft für Suchtforschung und Suchttherapie e.V. (DG-SUCHT) (2020) S3-Leitlinie: Screening, Diagnose und Behandlung alkoholbezogener Störungen (Langfassung)

[4] Conigrave KM, Degenhardt LJ, Whitfield JB, Saunders JB, Helander A, Tabakoff B (2002) CDT, GGT, and AST as markers of Alcohol Use: the WHO/ISBRA Collaborative Project. Alcohol Clin Exp Res 26:332–339. 10.1111/j.1530-0277.2002.tb02542.x 11923585

[5] Wurst FM, Thon N, Yegles M, Schrück A, Preuss UW, Weinmann W (2015) Ethanol metabolites: their role in the assessment of alcohol intake. Alcohol Clin Exp Res 39:2060–2072. 10.1111/acer.12851 26344403 10.1111/acer.12851

[6] Isaksson A, Walther L, Hansson T, Andersson A, Alling C (2011) Phosphatidylethanol in blood (B-PEth): a marker for alcohol use and abuse. Drug Test Anal 3:195–200. 10.1002/dta.278 21438164 10.1002/dta.278

[7] Neumann J, Beck O, Helander A, Böttcher M (2020) Performance of PEth compared with other Alcohol biomarkers in subjects presenting for Occupational and Pre-employment Medical Examination. Alcohol Alcohol Oxf Oxfs 55:401–408. 10.1093/alcalc/agaa027 10.1093/alcalc/agaa027 PMC7338721 32363383

[8] Luginbühl M, Van Uytfanghe K, Stöth F, Wurst FM, Stove CP (2022) Current evolutions, applications, and challenges of phosphatidylethanol analysis for clinical and forensic purposes. WIREs Forensic Sci 4:e1456. 10.1002/wfs2.1456

[9] Hartmann S, Aradottir S, Graf M, Wiesbeck G, Lesch O, Ramskogler K, Wolfersdorf M, Alling C, Wurst FM (2007) Phosphatidylethanol as a sensitive and specific biomarker: comparison with gamma-glutamyl transpeptidase, mean corpuscular volume and carbohydrate-deficient transferrin. Addict Biol 12:81–84. 10.1111/j.1369-1600.2006.00040.x 17407500 10.1111/j.1369-1600.2006.00040.x

[10] Helander A, Péter O, Zheng Y (2012) Monitoring of the alcohol biomarkers PEth, CDT and EtG/EtS in an outpatient treatment setting. Alcohol Alcohol Oxf Oxfs 47:552–557. 10.1093/alcalc/ags065 10.1093/alcalc/ags065 22691387

[11] Kechagias S, Dernroth DN, Blomgren A, Hansson T, Isaksson A, Walther L, Kronstrand R, Kågedal B, Nystrom FH (2015) Phosphatidylethanol compared with other blood tests as a biomarker of moderate alcohol consumption in healthy volunteers: a prospective Randomized Study. Alcohol Alcohol Oxf Oxfs 50:399–406. 10.1093/alcalc/agv038 10.1093/alcalc/agv038 25882743

[12] Alling C, Gustavsson L, Månsson JE, Benthin G, Anggård E (1984) Phosphatidylethanol formation in rat organs after ethanol treatment. Biochim Biophys Acta 793:119–122. 10.1016/0005-2760(84)90060-2 6704410 10.1016/0005-2760(84)90060-2

[13] Kobayashi M, Kanfer JN (1987) Phosphatidylethanol formation via transphosphatidylation by rat brain synaptosomal phospholipase D. J Neurochem 48:1597–1603. 10.1111/j.1471-4159.1987.tb05707.x 3559569 10.1111/j.1471-4159.1987.tb05707.x

[14] Gnann H, Engelmann C, Skopp G, Winkler M, Auwärter V, Dresen S, Ferreirós N, Wurst FM, Weinmann W (2010) Identification of 48 homologues of phosphatidylethanol in blood by LC-ESI-MS/MS. Anal Bioanal Chem 396:2415–2423. 10.1007/s00216-010-3458-5 20127079 10.1007/s00216-010-3458-5

[15] Helander A, Zheng Y (2009) Molecular species of the alcohol biomarker phosphatidylethanol in human blood measured by LC-MS. Clin Chem 55:1395–1405. 10.1373/clinchem.2008.120923 19423735 10.1373/clinchem.2008.120923

[16] Varga A, Hansson P, Johnson G, Alling C (2000) Normalization rate and cellular localization of phosphatidylethanol in whole blood from chronic alcoholics. Clin Chim Acta Int J Clin Chem 299:141–150. 10.1016/S0009-8981(00)00291-6 10.1016/s0009-8981(00)00291-6 10900300

[17] Schröck A, Thierauf A, Wurst FM, Thon N, Weinmann W (2014) Progress in monitoring alcohol consumption and alcohol abuse by phosphatidylethanol. Bioanalysis 6:2285–2294. 10.4155/BIO.14.195 25383738 10.4155/bio.14.195

[18] Gnann H, Weinmann W, Thierauf A (2012) Formation of phosphatidylethanol and its subsequent elimination during an extensive drinking experiment over 5 days. Alcohol Clin Exp Res 36:1507–1511. 10.1111/j.1530-0277.2012.01768.x 22458353 10.1111/j.1530-0277.2012.01768.x

[19] Schröck A, Thierauf-Emberger A, Schürch S, Weinmann W (2017) Phosphatidylethanol (PEth) detected in blood for 3 to 12 days after single consumption of alcohol-a drinking study with 16 volunteers. Int J Legal Med 131:153–160. 10.1007/s00414-016-1445-x 27596747 10.1007/s00414-016-1445-x

[20] Javors MA, Hill-Kapturczak N, Roache JD, Karns-Wright TE, Dougherty DM (2016) Characterization of the pharmacokinetics of Phosphatidylethanol 16:0/18:1 and 16:0/18:2 in human whole blood after Alcohol Consumption in a clinical laboratory study. Alcohol Clin Exp Res 40:1228–1234. 10.1111/acer.13062 27130527 10.1111/acer.13062 PMC4939838

[21] Hill-Kapturczak N, Dougherty DM, Roache JD, Karns-Wright TE, Javors MA (2018) Differences in the synthesis and elimination of Phosphatidylethanol 16:0/18:1 and 16:0/18:2 after acute doses of Alcohol. Alcohol Clin Exp Res 42:851–860. 10.1111/acer.13620 29505133 10.1111/acer.13620 PMC5915873

[22] Brenner-Hartmann J, Fastenmeier W, Graw M (2022) Urteilsbildung in der Fahreignungsbegutachtung: Beurteilungskriterien, Überarbeitete und erweiterte 4. Auflage. Kirschbaum Verlag, Bonn

[23] Ulwelling W, Smith K (2018) The PEth Blood Test in the security environment: what it is; why it is important; and interpretative guidelines. J Forensic Sci 63:1634–1640. 10.1111/1556-4029.13874 30005144 10.1111/1556-4029.13874

[24] Luginbühl M, Wurst FM, Stöth F, Weinmann W, Stove CP, Van Uytfanghe K (2022) Consensus for the use of the alcohol biomarker phosphatidylethanol (PEth) for the assessment of abstinence and alcohol consumption in clinical and forensic practice (2022 Consensus of Basel). Drug Test Anal 14:1800–1802. 10.1002/dta.3340 35851997 10.1002/dta.3340

[25] Faller A, Richter B, Kluge M, Koenig P, Seitz HK, Thierauf A, Gnann H, Winkler M, Mattern R, Skopp G (2011) LC-MS/MS analysis of phosphatidylethanol in dried blood spots versus conventional blood specimens. Anal Bioanal Chem 401:1163–1166. 10.1007/s00216-011-5221-y 21743983 10.1007/s00216-011-5221-y

[26] Luginbühl M, Weinmann W, Butzke I, Pfeifer P (2019) Monitoring of direct alcohol markers in alcohol use disorder patients during withdrawal treatment and successive rehabilitation. Drug Test Anal 11:859–869. 10.1002/dta.2567 30618164 10.1002/dta.2567

[27] Bantle M, Van Tieghem L, Weinmann W, Luginbühl M (2023) Lyso-Phosphatidylethanol detected by LC-MS/MS as a potential new marker for alcohol consumption. Eur J Mass Spectrom 29:338–347. 10.1177/14690667231200143 10.1177/14690667231200143 37709266

[28] Stöth F, Koch K, Bantle M, Pütz P, Wortmann F, Weinmann W (2023) Increase of PEth after single consumption of Alcohol and evaluation of a volumetric DBS Filter Paper device. J Anal Toxicol 47:379–384. 10.1093/jat/bkad009 36790103 10.1093/jat/bkad009

[29] Luginbühl M, Weinmann W, Al-Ahmad A (2017) Introduction of sample tubes with sodium azide as a preservative for ethyl glucuronide in urine. Int J Legal Med 131:1283–1289. 10.1007/s00414-017-1633-3 28712037 10.1007/s00414-017-1633-3

[30] Herzog J, Skopp G, Musshoff F, Hartung B (2023) Formation of phosphatidylethanol and ethylglucuronide after low to moderate alcohol consumption in volunteers with a previous three-week alcohol abstinence. Alcohol Alcohol 58:599–605. 10.1093/alcalc/agad025 37097639 10.1093/alcalc/agad025

[31] Stöth F, Kotzerke E, Thierauf-Emberger A, Weinmann W, Schuldis D (2023) Can PEth be detected with a cutoff of 20 ng/mL after single alcohol consumption? J Anal Toxicol 46:e232–e238. 10.1093/jat/bkac069 36107736 10.1093/jat/bkac069

[32] Perilli M, Toselli F, Franceschetto L, Cinquetti A, Ceretta A, Cecchetto G, Viel G (2023) Phosphatidylethanol (PEth) in blood as a marker of unhealthy alcohol use: a systematic review with Novel Molecular insights. Int J Mol Sci 24. 10.3390/ijms241512175 10.3390/ijms241512175 PMC10418704 37569551

[33] Aboutara N, Jungen H, Szewczyk A, Müller A, Iwersen-Bergmann S (2023) PEth 16:0/18:1 and 16:0/18:2 after consumption of low doses of alcohol-A contribution to cutoff discussion. Drug Test Anal 15:104–114. 10.1002/dta.3376 36181234 10.1002/dta.3376

[34] Magnusson B, Näykki T, Hovind H, Krysell M (2012) Handbook for calculation of measurement uncertainty in environmental laboratories, 3.1. Nordtest TR 537

[35] Paul L, Mußhoff F (2009) Richtlinie Der GTFCh Zur Qualitätssicherung bei forensisch-toxikologischen Untersuchungen. SoTaFC GTFCh

[36] Zheng Y, Beck O, Helander A (2011) Method development for routine liquid chromatography-mass spectrometry measurement of the alcohol biomarker phosphatidylethanol (PEth) in blood. Clin Chim Acta Int J Clin Chem 412:1428–1435. 10.1016/j.cca.2011.04.022 10.1016/j.cca.2011.04.022 21531215

[37] Francke MI, Peeters LEJ, Hesselink DA, Kloosterboer SM, Koch BCP, Veenhof H, De Winter BCM (2022) Best practices to implement dried blood spot sampling for therapeutic drug monitoring in clinical practice. Ther Drug Monit 44:696–700. 10.1097/FTD.0000000000000994 35607881 10.1097/FTD.0000000000000994 PMC9467683

[38] Capiau S, Wilk LS, De Kesel PMM, Aalders MCG, Stove CP (2018) Correction for the Hematocrit Bias in dried blood spot analysis using a nondestructive, single-wavelength reflectance-based hematocrit prediction method. Anal Chem 90:1795–1804. 10.1021/acs.analchem.7b03784 29281263 10.1021/acs.analchem.7b03784

